# New Insights into Iodide Transport Defects (ITDs) from the Characterization of a Heterozygous NIS Missense Variant (p.G288S) Identified in a Family with Thyroid Dysfunction During Pregnancy

**DOI:** 10.3390/ijms27125160

**Published:** 2026-06-06

**Authors:** Maddi Garate-Etxeberria, Mari Paz Lopez-Molina, Rafael Hortiguela, Pouya Alikhani, María De la Calle, Custodia García-Jimenez, Jose Carlos Moreno, Antonio De la Vieja

**Affiliations:** 1 Tumor Endocrine Unit, Chronic Disease Program (UFIEC), Instituto de Salud Carlos III, Ctra. Majadahonda a Pozuelo km 2.2, 28220 Madrid, Spain; maddi.garate@isciii.es (M.G.-E.);; 2 Thyroid Molecular Laboratory, Institute for Medical and Molecular Genetics (INGEMM), La Paz University Hospital Research Institute (IdiPAZ), 28029 Madrid, Spainjosecarlos.moreno@salud.madrid.org (J.C.M.); 3 Department of Obstetrics and Gynecology, La Paz University Hospital, 28046 Madrid, Spain; 4 Area of Physiology, Faculty of Health Sciences, University Rey Juan Carlos (URJC), 28922 Madrid, Spain; custodia.garcia@urjc.es; 5 U-753 The Rare Diseases Networking Biomedical Research Centre (CIBERER), Instituto de Salud Carlos III, 28029 Madrid, Spain

**Keywords:** iodide transport defect, sodium/iodide symporter, *SLC5A5*, thyroid dysfunction during pregnancy, heterozygous variant, NIS trafficking, iodide affinity, NIS dimerization

## Abstract

The Na^+^/I^−^ symporter (NIS) is the plasma membrane (PM) protein that actively mediates iodide (I^−^) transport into the thyroid gland. Pathogenic variants in the *SLC5A5* gene cause iodide transport defects (ITDs). A heterozygous G288S NIS variant was identified in a Spanish family in which female carriers developed thyroid dysfunction during pregnancy. Here, we characterized the functional significance of the G288S variant and other substitutions at residue 288 of human NIS. Human NIS (hNIS) expression and maturation were analyzed by immunoblotting, its subcellular localization was analyzed by immunofluorescence and flow cytometry, and its activity was analyzed by radioiodide uptake assays. The G288S variant does not affect hNIS maturation, membrane trafficking, or I^−^ uptake capacity, but significantly reduces I^−^ affinity while preserving substantial transport activity. In contrast, substitutions introducing charged residues (arginine, aspartic acid, or glutamic acid) or proline severely disrupted NIS maturation, plasma membrane targeting, and iodide transport. Because the variant was identified in heterozygosity, we evaluated residue 288 substitutions under heterozygous-like conditions. Co-expression of the patient-derived G288S variant with WT NIS produced an intermediate apparent *K_m_* without reducing *V_max_* compared with WT, consistent with a modest co-expression-dependent kinetic effect rather than a strong dominant-negative mechanism. In contrast, the severely disruptive G288E substitution reduced cell-surface NIS expression under co-expression conditions, providing proof-of-principle evidence that severe alteration of residue 288 can impair NIS plasma membrane delivery. These findings highlight residue 288 as a key determinant of hNIS functionality and underscore the need to carefully evaluate heterozygous *SLC5A5*/NIS variants, as they may become clinically relevant under conditions of increased physiological iodine demand and contribute to partial iodide transport impairment.

## 1. Introduction

Iodine is an essential micronutrient required for the biosynthesis of thyroid hormones (THs) T3 (3,5,3′-tri-iodo-L-thyronine) and T4 (3,5,3′,5′-tetra-iodo-Lthyronine or thyroxine). THs regulate development, growth, metabolism, and cellular function in virtually all tissues [1]. Adequate iodide uptake is essential for preventing iodine deficiency disorders (IDDs), which can result in goiter and hypothyroidism at all ages. Severe iodide deficiency during fetal and early postnatal life can cause irreversible neurodevelopmental and growth impairment [2,3,4]. The World Health Organization (WHO) recommends a daily intake of 90 µg for preschool children (0–59 months), 120 µg for school-age children aged 6 to 12 years, and 150 µg for adolescents and adults. During pregnancy and lactation, a woman’s iodine requirement increases from 150 to 250 μg/day [4,5]. Since their daily requirements are higher, pregnant women face a greater risk of iodine insufficiency, which has been associated with miscarriage, stillbirth, preterm delivery, and fetal congenital abnormalities [3,6].

The sodium/iodide symporter (NIS), encoded by the *SLC5A5* gene, is the plasma membrane (PM) glycoprotein that mediates the active transport of iodide (I^−^) from the bloodstream into various tissues, including the thyroid gland, salivary glands, stomach, intestine, ovary, placenta, kidney, and lactating mammary glands [7,8]. NIS, located on the basolateral surface of thyroid follicular cells, couples the transport of two sodium ions (Na^+^) with one iodide ion (I^−^), using the electrochemical gradient of Na^+^ to drive I^−^ uptake against its concentration gradient [9,10].

NIS has been reported to form dimers and must be located at the basolateral membrane in order to be functional [11,12,13,14]. Experimental studies suggest that NIS may also adopt higher-order oligomeric states and that dimerization may contribute to NIS trafficking, maturation, and functional expression at the plasma membrane, although its precise physiological role has not yet been fully established [11,12]. However, the pathophysiological relevance of NIS oligomerization in the context of heterozygous disease-associated variants remains poorly understood.

NIS is a glycoprotein with 13 transmembrane segments (TMSs), an extracellular amino terminus, and an intracellular carboxyl terminus [15,16]. Recently, its three-dimensional structure was resolved at atomic resolution through single-particle cryo-electron microscopy, providing major mechanistic insight into the substrate recognition, ion coupling, and translocation of NIS [13]. These studies identified the I^−^-binding cavity and two Na^+^-binding sites and showed that local conformational rearrangements in the central transport core are critical for substrate binding and transport. Importantly, they also demonstrated that variants affecting residues lining this region can markedly alter the apparent affinity for I^−^ and Na^+^.

To date, more than 40 pathogenic variants in NIS have been identified, leading to various alterations affecting different levels of NIS synthesis, transport and function: biosynthesis, folding, glycosylation, plasma membrane targeting, and/or iodide transport activity [17]. The absence of functional NIS results in iodide transport defects (ITDs), which can lead to congenital hypothyroidism, goiter, and cognitive and physical disabilities [9,18]. Some biallelic *SLC5A5* defects are classically associated with congenital ITDs [19,20,21]; however, the clinical and mechanistic relevance of heterozygous variants remains poorly understood, particularly under conditions of increased physiological iodine demand. Pregnancy may represent a critical stress condition in which otherwise subclinical defects in iodide transport become clinically apparent.

In this study, we identified and characterized a novel heterozygous *SLC5A5* variant, c.862G>A (p.G288S), in three generations of a Spanish family presenting with thyroid dysfunction during pregnancy. To our knowledge, this is the first report of a heterozygous *SLC5A5*/NIS variant associated with clinically relevant iodide transport impairment that is unmasked during pregnancy. Our findings indicate that the G288S variant exhibits reduced iodide affinity, which may predispose individuals to ITDs during periods of increased iodine demand, such as pregnancy. Furthermore, our co-expression analyses indicate that residue 288 substitutions can affect NIS behavior under heterozygous-like conditions, with G288S producing a modest kinetic shift in iodide affinity and the severely disruptive G288E substitution providing proof-of-principle evidence for impaired plasma membrane delivery. Clinically, this highlights the potential for partial ITDs in heterozygous carriers. In conclusion, these results underscore the critical role of residue 288 in maintaining NIS function.

## 2. Results

### 2.1. Case Report

**Figure 1:**
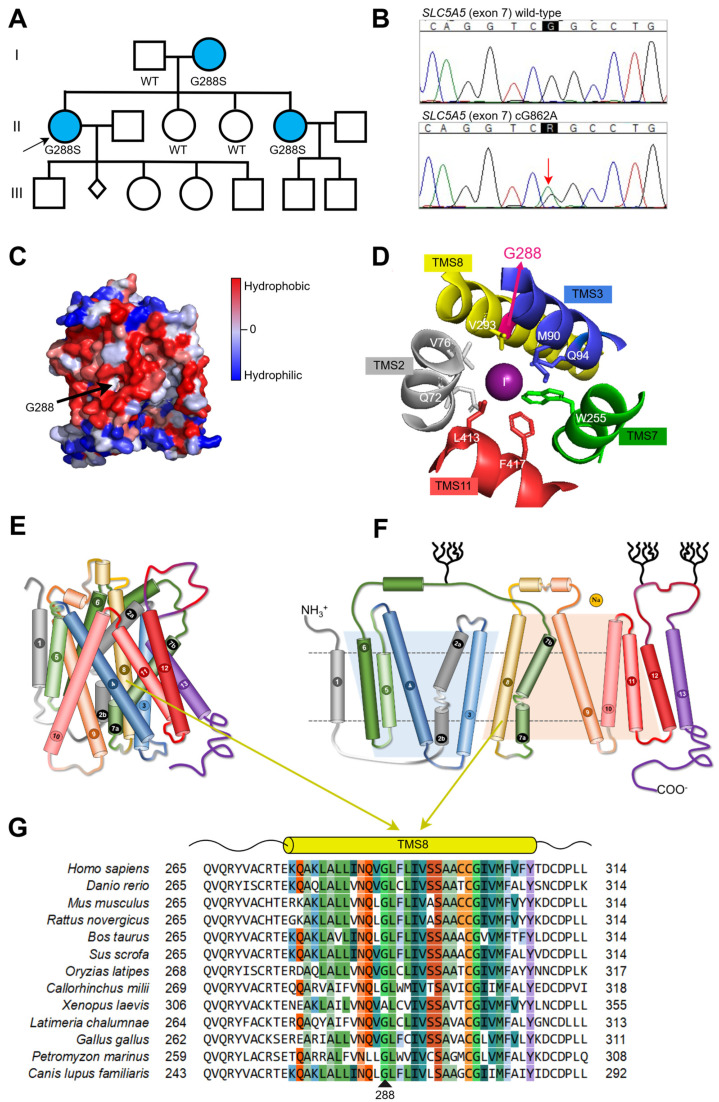
Genetic, structural, and evolutionary analysis of the *SLC5A5* p.G288S variant. (**A**) Segregation analysis of the *SLC5A5* variant p.G288S in three generations of a family with gestational hypothyroidism and goiter. The variant segregates with thyroid abnormalities in female carriers, including gestational hypothyroidism with a return to euthyroid status post-pregnancy in generation II and multinodular goiter in generation I. The index patient is indicated by the black arrow. (**B**) Chromatograms display the wild-type *SLC5A5* exon 7 sequence and the heterozygous c.862G>A variant. (**C**) Hydrophobicity of NIS surface. Hydrophobicity scale was calculated according to the Kyte–Doolittle scale, which ranges from –4.5 (blue, highly hydrophilic) to +4.5 (red, highly hydrophobic), with white representing neutral values (0.0). Calculations and visualization were performed using PyMOL v3.1.5.1 (Schrödinger, LLC, New York, NY, USA). Position G288 is highlighted. (**D**) Three-dimensional structure of rat NIS (PDB: 7UV0) highlighting residues that line the iodide-binding cavity and the location of G288 (top view). (**E**) 3D topology of hNIS based on the most recent three-dimensional structure [13]. (**F**) hNIS secondary structure model showing 13 TMSs (cylinders) and 3 glycosylation sites (branches). (**G**) Multiple sequence alignment of NIS orthologs from different species. Residues corresponding to transmembrane segment 8 (TMS8) are colored according to their physicochemical properties. The position corresponding to human residue G288 is indicated.

The G288S substitution was identified in three generations of a family from Spain with a history of thyroid abnormalities, including gestational hypothyroidism (Figure 1A). The index patient (II.1) was a 28-year-old woman diagnosed with gestational hypothyroidism during the first trimester of her first pregnancy. The diagnosis was based on elevated serum TSH levels (3.29 mU/L, reference range: <2.5 mU/L for the first trimester) and borderline low free thyroxine, FT4 (0.92 ng/dL, reference range: 0.93–1.70). Total T3 was within the normal range (1.25 ng/mL, reference range: 0.60–1.81). Free T3 was not available, as it was not measured during routine clinical follow-up [22]. Thyroglobulin levels were 36 ng/mL (r.r.: 0.6–60), and thyroid autoantibodies (anti-TPO and anti-TG) were negative.

Thyroid function tests were performed using electrochemical luminescent immunoassays on an Atelica IM Analyzer (Siemens Healthcare Diagnostics Inc., Tarrytown, NY, USA). Thyroid ultrasonography was performed during the second trimester and revealed a normally located, normally sized gland, with homogeneous echotexture and no nodular lesions. Each lobe measured 42 × 13 × 15 mm, corresponding to a total thyroid volume of 8.51 mL, which is within the normal range for adult women (75th percentile).

The patient was treated with low-dose levothyroxine (LT4; 25–50 µg/day, Eutirox) starting in the second trimester, resulting in normalization of TSH levels (1.89 and 1.49 mU/L at the second and third trimesters, respectively). After delivery, thyroid function returned to euthyroid status (TSH: 1.62 mU/L [r.r.: 0.27–4.2]; FT4: 1.08 ng/dL [r.r.: 0.93–1.7]), and LT4 therapy was discontinued.

The patient’s subsequent pregnancy resulted in early miscarriage. During her two subsequent pregnancies, hyperthyrotropinemia was again detected in the first trimester (TSH: 2.92 and 3.2 mU/L), and LT4 therapy was promptly initiated, achieving normalization of thyroid function. Neonatal screening of all offspring showed normal TSH values (<10 mU/L in filter-paper eluate).

### 2.2. Genetics

A heterozygous nucleotide substitution, c.862G>A, was detected in exon 7 of *SLC5A5* (RefSeq NM_000453.3), with the predicted the amino acid substitution being p.Gly288Ser (G288S). This variant corresponds to dbSNP entry rs142014480 (GRCh38 chr19:17,877,986G>A) (Figure 1B). An NGS panel including more than 320 genes related to thyroid disorders did not identify additional suspicious pathogenic or likely pathogenic variants in other genes involved in dyshormonogenesis or thyroid dysfunction in affected individuals from two generations of the pedigree (I.2 and II.1) [23].

The G288S variant was present in the index patient (II.1), in one sister (II.4) diagnosed with gestational hyperthyrotropinemia but returned to euthyroid status post-pregnancy, and their mother (I.2), who presented with euthyroid adult-onset multinodular goiter. The mother of the index patient (I.2) had not been evaluated with thyroid function tests during pregnancy, and therefore transient gestational hyperthyrotropinemia in this carrier cannot be excluded retrospectively. Among the tested relatives, the variant was absent in family members without the phenotype (Figure 1A).

In silico prediction tools supported a potentially deleterious effect (SIFT: 0.01, deleterious; PolyPhen-2: 0.765, possibly damaging; CADD PHRED: 26.9). These computational data support application of The American College of Medical Genetics and Genomics (ACMG) criterion PP3 at a supporting level for the functional relevance of the substitution [24,25]. The p.Gly288Ser variant is rare in population databases (allele frequency of approximately 5.1 × 10^−4^ in aggregated gnomAD datasets) and is currently classified as a variant of uncertain significance (VUS) in ClinVar [26].

According to the three-dimensional structure of rat NIS [13], which shares 89% sequence identity with human NIS, residue G288 is located within TMS8 and in a hydrophobic region (Figure 1C). G288 is found in proximity to several amino acid residues important for iodide binding (Figure 1D,F). This suggests that the G288S substitution could interfere with iodide binding and, consequently, protein function. Multiple sequence alignment of NIS orthologs showed that glycine 288 and its surrounding hydrophobic environment are highly conserved across vertebrate species (Figure 1G), supporting the functional relevance of this residue.

### 2.3. G288S NIS Reaches the Plasma Membrane but Exhibits Reduced Iodide Affinity Despite Preserved Transport Capacity

**Figure 2:**
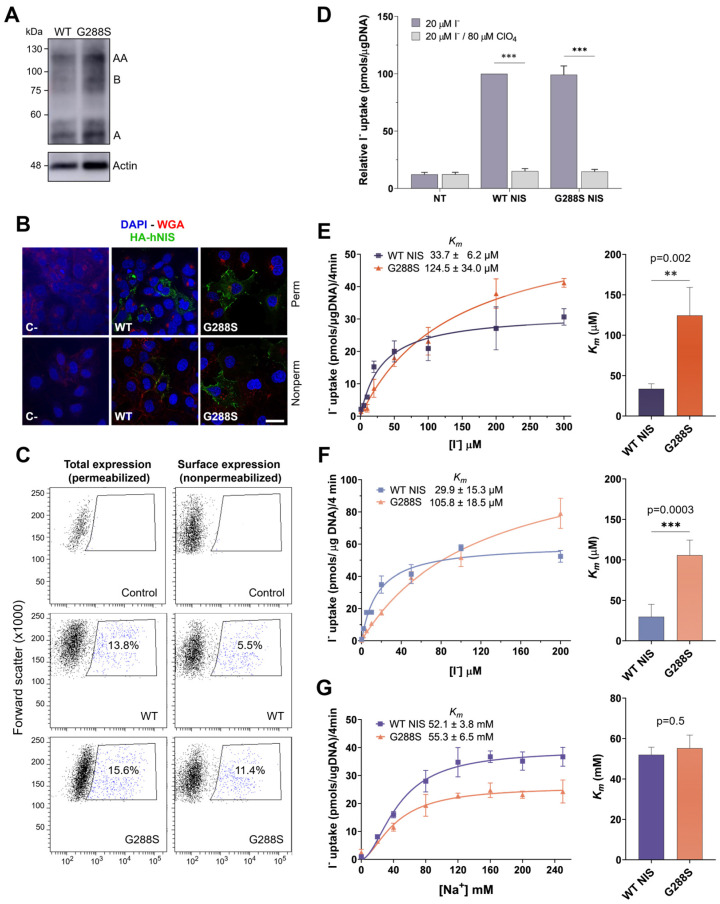
Characterization of human G288S NIS in transfected cells and kinetic analysis of I^−^ and Na+ dependence. (**A**) Immunoblot analysis of total protein extracts isolated from COS-7 cells transfected with either human WT or G288S NIS cDNA. Human NIS protein forms were detected using an anti-HA antibody (A, AA: ∼55 and ∼110 kDa, corresponding to core-glycosylated NIS and its dimer, respectively; B: ∼95kDa, corresponding to mature NIS protein). Lines on the left side of the blot indicate the relative electrophoretic mobilities of the corresponding NIS bands. Actin was used as the loading control. (**B**) Confocal imaging of COS-7 cells expressing similar levels of WT or G288S NIS under permeabilized and non-permeabilized conditions. Cells were immunostained with an anti-HA antibody followed by fluorescein-conjugated goat anti-mouse antibody. Scale bar is 30 μm in all images. (**C**) Flow cytometry analysis (FACS) using an anti-HA antibody to detect NIS expression in COS-7 cells at the cell surface (non-permeabilized cells) or anywhere within the cell (permeabilized). (**D**) Steady-state iodide transport assays. Cells, same as those in B and C, were incubated in the presence of 20 µM I^−^/140 mM Na^+^ or 20 µM I^−^/140 mM Na^+^/80 µM ClO4^−^. Each value represents the mean relative pmol I^−^/µg DNA from four independent experiments performed in triplicate. Statistical comparisons were performed using unpaired two-tailed Student’s *t*-test, with significance levels indicated as *** *p* < 0.001. (**E**) Kinetic analysis of iodide uptake in transfected COS-7 cells. Results are expressed as pmol I^−^/µg DNA ± SD. Values represent the average of at least three different experiments; in each experiment, activity was analyzed in triplicate. (**F**) Kinetic analysis of iodide uptake in MDCK II cells stably expressing either human NIS or G288S. Initial rates (4 min time point) of iodide uptake were determined and analyzed, similarly to panel (**E**). (**G**) Na^+^-dependent kinetic analysis. The corresponding apparent *K_m_* values and statistical comparisons are shown in the bar graphs, with *p* values indicated in the figure. The results are expressed as pmol I^−^/µg DNA ± SD from three independent experiments. Statistical comparisons were performed using an unpaired two-tailed Student’s *t*-test, with significance levels indicated as *** *p* < 0.001; ** *p* < 0.01.

To investigate the impact of the G288S substitution on NIS, we introduced either human WT or G288S NIS cDNA into COS-7 cells and performed immunoblot analysis on protein extracts obtained from the transfected cells. The expression of NIS protein was similar in cells transfected with WT and G288S NIS cDNA (Figure 2A). For both WT NIS and G288S NIS, we observed a glycosylated form at 95 kDa (B) and a non-fully glycosylated 55 kDa (A) form.

We thoroughly examined human G288S NIS targeting to the plasma membrane using cytometric analysis and confocal immunofluorescence microscopy. Confocal immunofluorescence observations of non-permeabilized cells demonstrated a distinct plasma membrane-associated immunofluorescent NIS staining pattern in COS-7 cells transfected with either WT or with G288S NIS cDNA constructs (Figure 2B). The immunofluorescence of permeabilized cells showed similar levels of protein expression for both WT and G288S.

Furthermore, flow cytometric analysis consistently revealed that G288S NIS was accurately directed to the plasma membrane, in line with our immunofluorescence findings, and displayed no differences compared to WT NIS protein (Figure 2C). These findings provide additional evidence that human G288S NIS is glycosylated and properly matured and targeted to the cell surface like WT NIS.

The iodide transport capacity was evaluated in cells transfected with WT NIS and G288S human NIS with and without perchlorate (ClO4^−^), a competitive NIS inhibitor. Steady-state measurements (Figure 2D) revealed no significant difference between cells expressing WT NIS and G288S NIS. However, kinetic studies revealed a higher apparent *K_m_* for I^−^ (124.52 ± 34.60 µM) for G288S NIS, contrasting with the apparent *K_m_* of WT NIS (33.73 ± 6.19 µM) (Figure 2E). This indicates a marked decrease in affinity for I^−^ for the G288S variant protein. These data suggest that residue 288 and, more broadly the structural region in which it is located, contributes to substrate recognition and/or transport coupling. To eliminate the experimental variation in transient transfection, we stably transfected MDCK cells with WT and G288S human NIS (Figure 2F). I^−^ transport kinetics in stable clones recapitulated those of COS-7 cells, with the *K_m_* of the G288S variant (105.81 ± 18.51 µM) being ~3.5-fold higher than that of WT NIS (29.86 ± 15.35 µM). Thus, although steady-state transport was preserved, kinetic analysis consistently demonstrated reduced apparent iodide affinity in G288S NIS in both transient and stable expression systems.

To determine whether this effect selectively involved iodide handling or also altered sodium dependence, we performed Na^+^-dependent kinetic analysis of WT and G288S NIS. The initial rates of iodide uptake were measured with increasing extracellular Na^+^ concentrations, and the data were fitted using an allosteric sigmoidal model (Figure 2G). No significant differences were detected between WT and G288S human NIS in terms of Na^+^-dependent kinetic parameters, indicating that the G288S substitution selectively affects apparent I^−^ affinity without significantly altering Na^+^ dependence.

### 2.4. Iodide Affinity and Plasma Membrane Expression Are Regulated by the Physicochemical Properties of Residue 288

**Figure 3:**
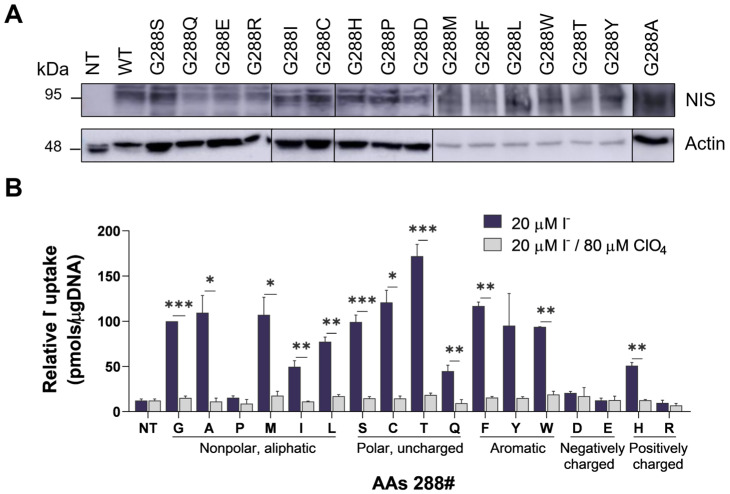
Characterization of substitutions at residues in position G288 in human NIS. (**A**) Immunoblot analysis. Total protein was isolated from non-transfected COS-7 cells (NT) and COS-7 cells transfected with cDNA of variants of human NIS carrying substitutions at residue 288. Human NIS protein forms were detected using an anti-hNIS-ETNL antibody. Actin was used as the loading control. (**B**) Steady-state iodide transport assays, same as in A. Each value represents the mean ± SEM of relative pmol I^−^/µg DNA from at least two independent experiments performed in triplicate. Statistical comparisons were performed using unpaired two-tailed Student’s *t*-test, with significance levels indicated as *** *p* < 0.001; ** *p* < 0.01; * *p* < 0.05.

To investigate the significance of position 288 in the human NIS protein, we explored the effects of substituting various residues at this location. Using site-directed mutagenesis, we introduced Ile, Leu, Phe, Cys, Met, Ala, Trp, Gly, Thr, Ser, Tyr, His, Pro, Asp, Gln, Glu, and Arg at position 288. Subsequently, all constructs were transiently transfected into COS-7 cells, and human NIS expression was analyzed by immunoblotting (Figure 3A).

#### 2.4.1. Certain Substitutions at Residue 288 Impair Plasma Membrane Targeting

**Figure 4:**
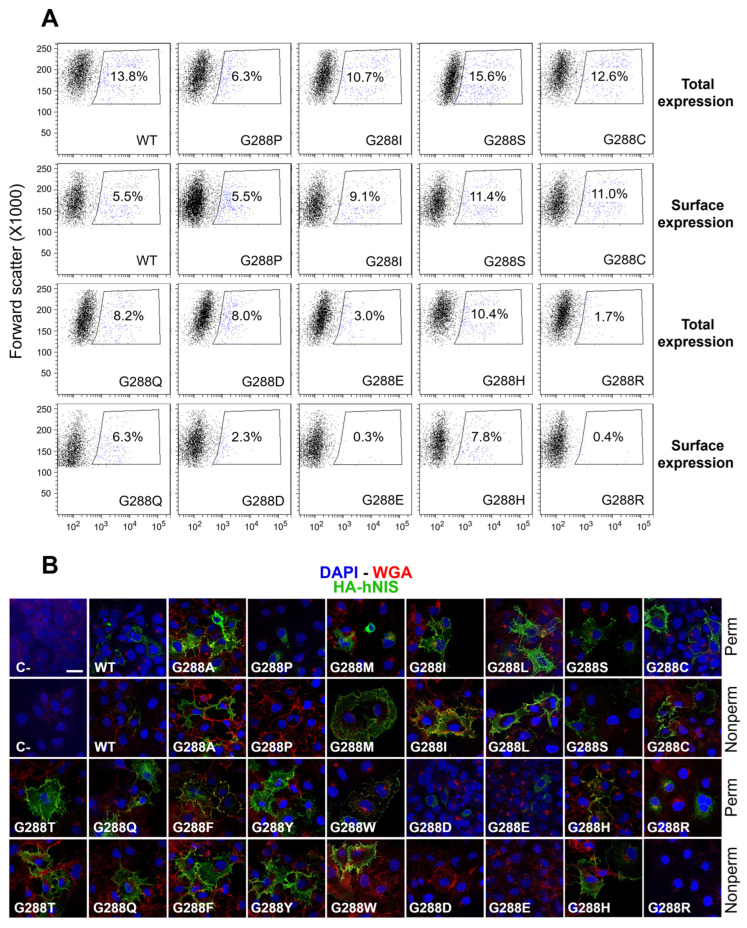
Cellular localization and surface expression of human NIS variants carrying substitutions at residue G288. (**A**) Flow cytometry analysis (FACS) of WT and NIS mutants. An anti-HA antibody was used to detect human NIS expression in COS-7 cells at the cell surface (non-permeabilized cells) or anywhere within the cell (permeabilized). (**B**) Confocal imaging of NIS G288 mutants using immunofluorescence in COS-7 cells under permeabilized and non-permeabilized conditions. Scale bar is 30 μm in all images.

Cells expressing G288P, G288D, G288E, or G288R did not show ClO4^−^-sensitive iodide transport (Figure 3B). Specifically, G288E failed to accumulate I^−^, even at high concentrations. FACS and immunofluorescence assays indicated that the deficiency in G288P, G288D, G288E, and G288R NIS can be attributed to impaired delivery to the cell surface (Figure 4). In contrast, other mutant proteins, featuring amino acids with uncharged side chains at position 288, reached the plasma membrane similarly to WT NIS (Figure 4) and facilitated significant levels of I^−^ transport (Figure 3B). These results indicate that certain substitutions at residue 288, particularly proline and charged residues, disrupt proper trafficking of human NIS to the cell surface and thereby abolish transport activity.

#### 2.4.2. Polar Substitutions at Residue 288 Are Associated with Reduced Apparent Iodide Affinity

**Figure 5:**
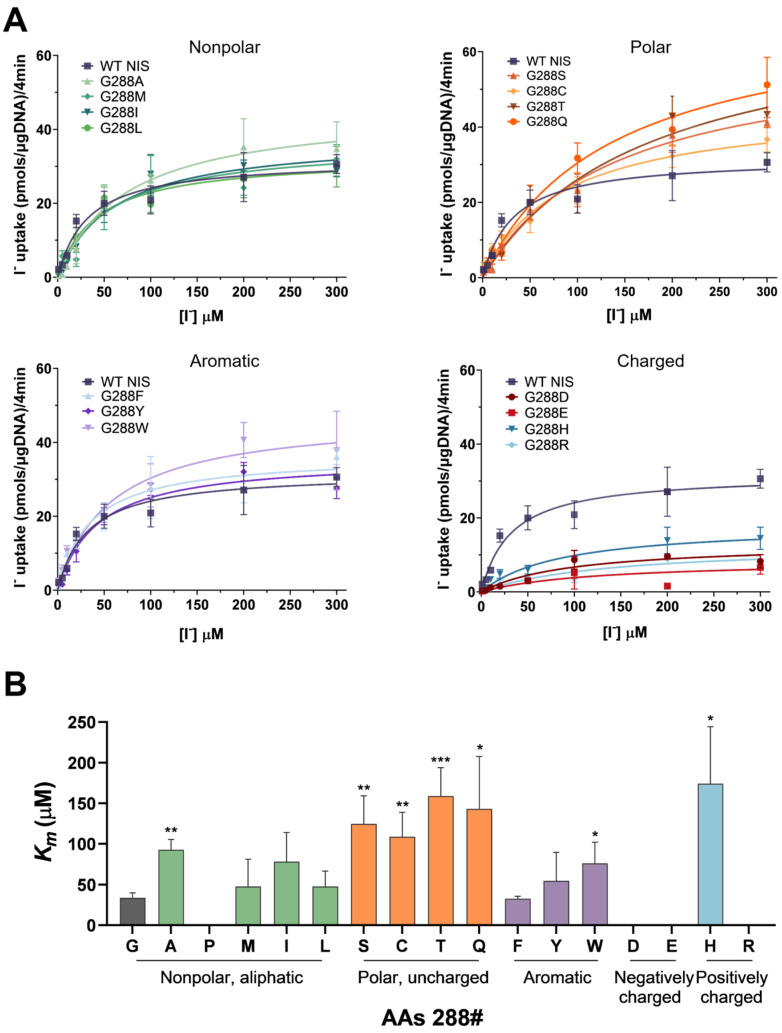
Kinetic analysis of iodide uptake in cells expressing human NIS mutants carrying substitutions at residue 288. (**A**) Representative kinetic curves showing initial rates (4 min time point) of iodide uptake determined at the indicated concentrations. The results are expressed as pmol I^−^/µg DNA ± SD. Values represent the average of at least three different experiments; in each experiment, activity was analyzed in triplicate. (**B**) Apparent *K_m_* values for each mutant normalized to that of WT. Data are shown as the mean ± SD from three independent experiments. Statistical comparisons were performed using unpaired two-tailed Student’s *t*-test, with significance levels indicated as *** *p* < 0.001; ** *p* < 0.01; * *p* < 0.05.

To understand how the residue at position 288 influences iodide uptake kinetics, we analyzed the kinetic properties of iodide uptake in COS-7 cells expressing all described NIS mutants. The initial rates were measured by assessing iodide accumulation at 4 min time points over a range of iodide concentrations (1–300 µM), with a constant 140 mM Na^+^ concentration (Figure 5A). Kinetic analysis of mutants that reached the plasma membrane (those carrying substitutions at residue 288 with amino acids with nonpolar side chains) showed a *K_m_* similar to that of WT NIS. In contrast, mutants carrying substitutions with polar side chains, such as G288C, G288T, or G288Q, exhibited a significantly higher *K_m_* compared to WT NIS. Aromatic substitutions produced intermediate effects. Finally, for mutants with proline or charged side chains (G288P, G288D, G288E, and G288R), it was not possible to detect iodide uptake or measure its kinetics. This is consistent with the lack of protein expression at the plasma membrane described above (Figure 4). A comparison of the *K_m_* variations for all these mutants is presented in Figure 5B.

Collectively, these findings establish that the physicochemical nature of residue 288 is crucial for proper human NIS expression at the plasma membrane and its optimal activity. More specifically, the data indicate that both trafficking to the plasma membrane and apparent iodide affinity are strongly influenced by the physicochemical properties of the residue at position 288, with polar substitutions generally associated with higher apparent *K_m_* values and charged or proline substitutions frequently impairing cell-surface expression and transport activity. These findings suggest that substitution at position 288 in human NIS protein could result in partial or complete ITDs.

### 2.5. G288E Affects NIS Cell-Surface Delivery, Whereas G288S Produces a Partial Kinetic Defect Under Heterozygous-like Conditions

Since the G288S variant was identified in a heterozygous state and since NIS has been reported to form dimers or higher-order oligomers, we next examined whether substitutions at residue 288 could affect NIS function or its expression on the cell surface under conditions similar to those of the heterozygous state. First, we used the G288E substitution, which causes a severe disorder, as a proof-of-concept model to test whether a mutation at residue 288 that disrupts transport could interfere with NIS transport to the plasma membrane. We then directly evaluated the patient-derived G288S variant under co-expression conditions.

**Figure 6:**
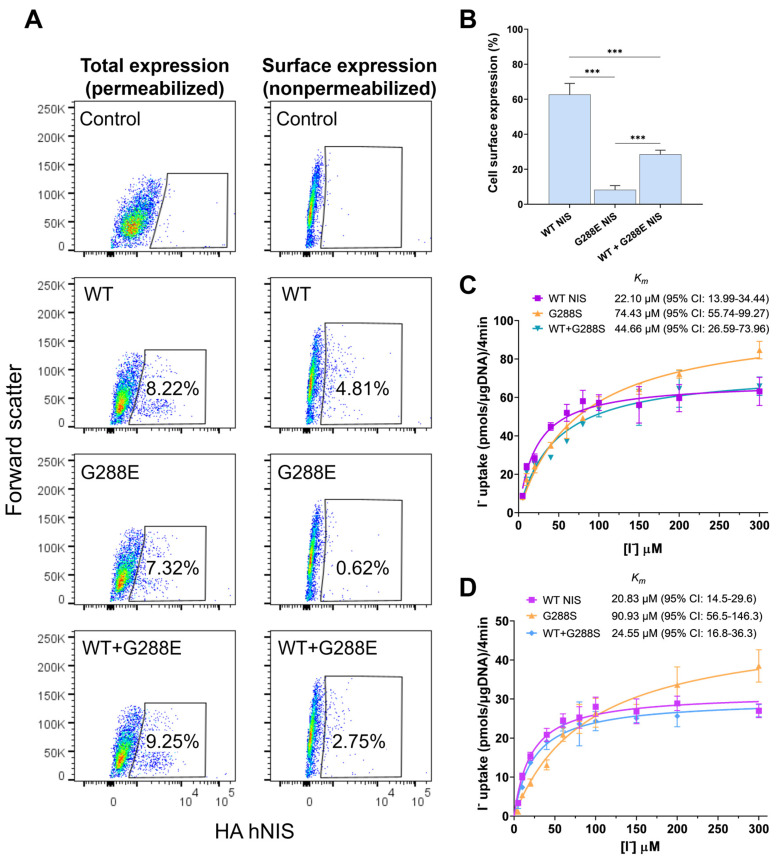
Subcellular location and activity of NIS variants under single-expression and co-expression conditions (homozygous-like vs. heterozygous-like status). (**A**) Representative flow cytometry analysis (FACS) of COS-7 cells transfected with either empty plasmid (5 µg), WT human NIS (2.5 µg WT cDNA + 2.5 µg empty plasmid), human G288E NIS (2.5 µg G288E cDNA + 2.5 µg empty plasmid), or both (2.5 µg G288E cDNA + 2.5 µg WT NIS cDNA). NIS expression was detected at the cell surface (non-permeabilized cells) or throughout the cell (permeabilized cells) using an anti-HA antibody. A representative graph from 3 independent experiments is shown. (**B**) Surface expression in plasma membrane. Values were corrected for background fluorescence using cells transfected with empty plasmid. Data are shown as the mean ± SD from three independent experiments. Statistical comparisons were performed using unpaired two-tailed Student’s *t*-test, with significance levels indicated as *** *p* < 0.001. (**C**) Kinetic analysis of iodide uptake in COS-7 cells transfected with WT NIS alone, G288S NIS alone, or both WT and G288S NIS. (**D**) Kinetic analysis of iodide uptake in mixed populations of MDCK cells expressing WT NIS and G288S NIS. Transport activity was normalized to the NIS expression levels determined by Western blot. The kinetic results are expressed as pmol I^−^/µg DNA ± SD. Values represent the average of quadruplicates. *K_m_* estimates are shown with 95% CIs.

Since charged amino acids such as G288E failed to accumulate iodide and did not reach the cell surface, we sought to determine whether the G288E variant could reduce the plasma membrane expression of the WT form of human NIS. We transiently transfected COS-7 cells with both WT NIS and G288E human NIS cDNA, all carrying an N-terminal HA tag to allow for detection with the anti-HA antibody under non-permeabilized conditions. Both hNIS variants were transfected individually and together to simulate situations of homozygosity and heterozygosity, respectively. Flow cytometric analysis of three independent experiments showed that the cells with hNIS expression under permeabilized conditions showed comparable mean values for WT (10.6%), G288E (8.52%) and WT+G288E (16.54%) expression (Figure 6A). In contrast, cell-surface hNIS expression, assessed under non-permeabilized conditions, was markedly reduced in WT+G288E co-transfected cells compared to WT-transfected cells. In cells transfected with WT hNIS, 6.67% of cells showed surface expression, whereas transfection with G288E alone resulted in almost no expression, and WT+G288E co-expression was observed in 4.64% of cells (Figure 6A, right panels). A comparison of cell-surface expression levels of WT or G288E NIS in homozygosity or the heterozygosity situation (WT+G288E) showed a reduction of more than 50% relative to WT alone (Figure 6B).

These findings indicate that, under WT+G288E co-expression conditions, the total cell-surface NIS signal is reduced compared with WT transfection alone. Because G288E is not the patient-derived variant and represents a more severe trafficking-defective substitution, these data should be interpreted as proof-of-principle evidence that severe alteration of residue 288 can impair NIS plasma membrane delivery, rather than as direct evidence for the mechanism of G288S. Importantly, total hNIS expression was not reduced under co-expression conditions, indicating a selective defect in cell-surface delivery rather than a global decrease in hNIS abundance. This observation is compatible with intermolecular interference between NIS molecules, potentially related to NIS oligomerization, although alternative explanations such as competition for folding or trafficking machinery, altered intracellular retention, indirect effects on plasma membrane delivery, or overexpression-related effects cannot be excluded. Together with the patient-derived G288S kinetic phenotype, these findings support the concept that heterozygous alterations affecting residue 288 may contribute to partial iodide transport impairment under conditions of increased iodide demand or limited iodide availability, compatible with a partial ITD-like phenotype.

We then directly evaluated the patient-derived G288S variant under conditions of heterozygous-like co-expression (Figure 6C). COS-7 cells were transfected with WT+empty vector, G288S+empty vector, or WT+G288S at a 1:1 plasmid ratio, while keeping the total DNA content constant. WT NIS displayed an apparent *K_m_* of 22.10 µM, whereas G288S showed a markedly higher *K_m_* of 74.43 µM. WT+G288S co-expression resulted in an intermediate *K_m_* of 44.66 µM. The *V_max_* values were 68.24 for WT, 100.7 for G288S, and 74.42 for WT+G288S. Analysis of *K_m_* values obtained from independent biological replicates showed significant global differences between conditions. Post hoc analysis showed that G288S differed significantly from WT and from WT+G288S, whereas the difference between WT and WT+G288S did not reach statistical significance after multiple-comparison correction. *V_max_* was not reduced in WT+G288S compared with WT. Thus, true co-expression of WT and G288S produced a modest kinetic shift toward lower apparent iodide affinity, without reducing the maximal transport capacity.

As a complementary approach, we mixed stable MDCK cell populations expressing either WT NIS or G288S NIS at defined ratios. This experiment does not represent true co-expression within the same cell, but provides an additional reference for the contribution of independent WT- and G288S-expressing cell populations. WT-only and G288S-only stable cell populations displayed apparent *K_m_* values of 20.83 µM and 90.93 µM, respectively. Mixed populations showed apparent *K_m_* values close to those of WT-expressing cells (24.55 µM). Analysis of replicate-derived *K_m_* values showed significant global differences among conditions (ANOVA, *p* = 0.0189). Tukey post hoc analysis showed that G288S-expressing cells differed significantly from WT-expressing cells and from both mixed populations, whereas neither mixed population differed significantly from the WT-expressing population. These data indicate that, when WT- and G288S-expressing cells are present as separate populations, the higher-affinity WT component predominates in the apparent kinetic profile.

Taken together, these experiments suggest that substitutions at residue 288 may affect NIS function through different mechanisms, depending on the physicochemical severity of the substitution and the cellular context. The G288E substitution, which causes a severe alteration, impairs NIS expression on the cell surface under co-expression conditions, providing a proof of principle that alterations at residue 288 can interfere with NIS transport to the plasma membrane. In contrast, the patient-derived G288S variant primarily causes a kinetic defect characterized by reduced apparent affinity for iodide. In co-expression experiments in COS-7 cells, WT+G288S exhibited an intermediate apparent *K_m_*, consistent with a moderate reduction in apparent iodide affinity that is dependent on co-expression. However, *V_max_* was not reduced compared to WT, and the difference in *K_m_* between WT and WT+G288S did not reach statistical significance after correction for multiple comparisons. Therefore, these data do not support a strong dominant-negative effect of G288S, but suggest a partial kinetic effect that could become relevant under conditions of increased iodine demand.

## 3. Discussion

The variant G288S human NIS was identified in a Spanish family in which heterozygous carriers developed thyroid dysfunction during pregnancy, which was diagnosed as gestational hypothyroidism. In contrast to classical ITD cases associated with NIS variants such as T354P, Y348D, or Q267E, which typically present with congenital or neonatal hypothyroidism, the G288S variant is associated with a milder phenotype, manifesting as gestational hyperthyrotropinemia with a return to euthyroid status after pregnancy. The clinical variability observed among heterozygous carriers may be due to the influence of several factors. In the affected carriers analyzed, who came from two generations, an NGS panel covering more than 320 thyroid-related genes did not identify any variants suspected of being pathogenic or likely pathogenic in other genes involved in dyshormonogenesis or thyroid dysfunction. However, other types of non-coding variants; polygenic backgrounds; differences in thyroid reserve, iodine intake or status; and environmental factors cannot be ruled out. Iodine status is particularly relevant, as pregnancy increases iodine requirements and renal clearance of iodide. Although Spain is currently considered a country with sufficient iodine intake at the population level, individual iodine intake can vary, and pregnant women remain a vulnerable group. Furthermore, the index patient’s mother was not evaluated with thyroid function tests during pregnancy, so transient gestational thyroid dysfunction in the previous generation cannot be ruled out. Her adulthood-onset euthyroid multinodular goiter could represent a late manifestation within the broader spectrum of partial NIS dysfunction, which could reflect chronic low-grade stimulation of a thyroid gland with impaired iodide handling.

Overall, this clinical pattern suggests that iodide transport capacity may be sufficient under baseline physiological conditions but becomes inadequate when iodine requirements and thyroid hormone demand increase, such as during pregnancy.

ITDs are typically associated with NIS pathogenic variants that follow a recessive inheritance pattern, and in some cases, two heterozygous variants are identified that together result in the disorder [27,28,29,30]. However, in the present case, the patient exhibited a clear clinical phenotype despite carrying the variant in a heterozygous state and lacking additional pathogenic variants. This scenario differs from previously reported biallelic or compound heterozygous *SLC5A5*/NIS cases [29,31,32] in which two pathogenic variants, one in each allele, are required to produce overt ITDs. In contrast, our findings suggest that a single heterozygous variant may become clinically relevant under conditions of increased physiological demand.

In this study, we characterized the molecular properties of the G288S NIS variant and examined the structural and functional relevance of residue 288 for NIS activity and proper targeting to the plasma membrane.

While the G288S NIS variant displays proper plasma membrane localization and maintains iodide transport activity at levels comparable to those observed with the WT protein (Figure 2B–D), closer analysis revealed subtle but functionally significant differences. Specifically, the G288S variant exhibited a marked reduction in iodide affinity (Figure 2E,F). Similar partial functional impairments have also been observed in other NIS variants, such as Q267E [27], which reaches the plasma membrane and retains transport activity, although at lower levels and with a decreased affinity compared to WT. Pathogenic variants of this type are unlikely to cause clinically relevant consequences under conditions of normal or high dietary iodide intake since the activity of the transporter is sufficient to sustain thyroid hormone synthesis. However, in situations of increased iodine demand, such as pregnancy, or in the context of iodine deficiency, these subtle alterations may become physiologically significant and lead to hypothyroidism or ITDs. In this regard, the approximately 3.5- to 3.7-fold increase in apparent *K_m_* observed for G288S is consistent with a partial kinetic defect that becomes clinically relevant only under conditions of increased physiological stress.

The iodide concentration range used in our kinetic assays was selected to define apparent *K_m_* and *V_max_* rather than to reproduce a single physiological plasma iodide concentration. Human plasma inorganic iodide concentrations have been reported to range from approximately 2 to 6 µg/L for the usual iodine intake (below 200 µg/day), which corresponds to 0.016–0.047 µM [33,34]. Therefore, physiological circulating inorganic iodide concentrations are far below the apparent *K_m_* values measured in vitro, and the upper iodide concentrations used in our assays should be considered supraphysiological concentrations required for kinetic modeling. Under physiologically low substrate concentration, NIS-mediated transport is expected to depend strongly on the *V_max_*/*K_m_* relationship. Accordingly, the increased apparent *K_m_* of G288S, despite preserved maximal transport capacity, would be expected to reduce the apparent transport efficiency, particularly when iodide availability is limited or the iodine demand is increased. This is especially relevant during pregnancy, when iodine requirements increase because of increased maternal thyroid hormone production, renal iodine losses, and fetal iodine requirements [22].

Although the NIS variant G288S is unlikely to cause overt hypothyroidism under baseline conditions, preserving the nonpolar environment around residue G288 within TMS8 is essential for NIS function, proper trafficking to the plasma membrane, and, consequently, for adequate TH synthesis. Phylogenetic analysis of different species revealed that the glycine residue at position 288 is highly conserved (Figure 1G). This evolutionary conservation suggests that alterations at this position are likely to have significant effects on protein conformation, trafficking, and iodide transport. In fact, polar residues at this position (G288S, G288C, G288T, and G288Q) reduced the apparent iodide affinity of NIS (Figure 5B). Side-chain characteristics are critical at multiple residues within NIS. For instance, the substitution of glycine with a polar amino acid has been shown to result in reduced iodide transport by NIS. A relationship with side-chain size has also been observed, with larger residues correlating with lower iodide transport efficiency [35,36]. We demonstrated that changes in the physicochemical properties of this region not only affect iodide affinity and transport, but also the proper trafficking of the protein to the plasma membrane. Indeed, variants carrying residues with charged side chains (either negatively or positively charged) (G288D, G288E, and G288R) or proline (an amino acid with a highly disruptive conformational effect) at position 288 fail to reach the plasma membrane and, therefore, completely lose their ability to transport iodide (Figure 3 and Figure 4). Although proline is a nonpolar amino acid, its rigid cyclic structure makes it highly disruptive [37,38,39]. The presence of proline at several positions in NIS has been shown to impair NIS functionality. Charge has also been described as a disruptive factor for NIS functionality in several studies [7,27,31,35,40,41,42]. The relatively preserved function of G288H can be explained by the unique properties of histidine. Although histidine can carry a positive charge, its imidazole side chain has a pKa close to physiological pH and is only partially protonated at pH 7.4. Furthermore, in the hydrophobic environment of TMS8, the neutral state may be favored, preventing the introduction of a permanent charge into the core of the transmembrane segment. The imidazole ring may also be accommodated through local hydrogen-bond interactions. This could explain why G288H behaves differently from substitutions with permanently charged residues, such as arginine, aspartate, or glutamate, which are more likely to disrupt the local electrostatic environment, folding, trafficking, or conformational dynamics necessary for NIS function. For example, the presence of a negatively charged residue at position 270 or 543 leads to intracellular retention of NIS, likely because the amino-acid charge interferes with proteins involved in its trafficking [28,35]. Likewise, specific leucine-containing motifs have also been shown to be critical for NIS trafficking [43,44]. Koumarianou et al. [44] demonstrated that the AP-1 adaptor complexes mediate basolateral NIS sorting through recognition of residues L121, LL562/563, and L583, underscoring the importance of leucine-based trafficking determinants for proper plasma membrane targeting of NIS.

G288 is located within TMS8, in a hydrophobic region (Figure 1C). It lies close to the iodide binding site; in fact, residue V293, which is part of TMS8, directly participates in iodide coordination [13]. Consistent with this structural arrangement, variants at G288 altered apparent iodide affinity, whereas no significant changes in Na^+^-dependent kinetic parameters were detected in our functional assays (Figure 2E–G). These findings support the idea that substitutions at position 288 primarily affect iodide handling rather than sodium interaction. Certainly, G288 is not located within any of the defined ion-binding sites of NIS [13]. Nevertheless, other residues located outside regions initially considered essential for NIS function have also been reported to be critical pathogenic variants, such as G543E [35]. This suggests that amino acids not involved in the canonical binding sites may still play key roles in maintaining the structural integrity and proper activity of NIS. Importantly, not only are residues contributing to ion binding critical, but also those that are involved in ion translocation and/or the final release of ions into the extra- or intra-cellular milieu. Consistent with the recently published three-dimensional structure, ion translocation occurs through a hydrophobic pore, and alterations in this region are likely to affect both transport efficiency and, in some cases, protein trafficking. Variants at the G288 residue could alter the local geometry or physicochemical environment of the transport core, thereby reducing the efficiency of ion translocation and/or release into the cell. This hypothesis is supported by the increased apparent *K_m_* observed for G288S and other mutants and by structural data from Ravera et al. [13], which suggests that L289 participates in the theoretical ion release tunnel.

In line with this concept, NIS variants containing nonpolar residues at position 288 exhibit proper PM trafficking and preserved protein function. We hypothesize that substitutions with polar amino acids at position 288 affect neighboring residues, thereby altering the local conformation and, consequently, iodide transport. This idea is supported by our finding that polar residues at position 288 interfere with NIS activity (Figure 5). This impairment is even more pronounced when the substitution involves charged amino acids (regardless of the polarity of the charge) or highly disruptive residues such as proline. Thus, our data supports a model in which the physicochemical nature of residue 288 influences both structural compatibility with the membrane-embedded transport core and the efficiency of iodide translocation.

The pathogenic variants described so far have been reported either in homozygosity or in compound heterozygosity with other NIS variants. Indeed, family members carrying heterozygous variants, with one normal allele, were not clinically affected [29]. This has been confirmed in vitro, where the function of the WT protein was not impaired when co-transfected with mutant alleles [28,29,45]. However, here, we report a family with a heterozygous variant who presented with clinical manifestations.

Because the G288S variant was identified in heterozygosity, we evaluated the behavior of residue 288 substitutions under heterozygous-like conditions (Figure 6). The severely disruptive G288E substitution reduced cell-surface NIS expression when co-expressed with WT NIS, indicating that alterations at residue 288 can impair NIS plasma membrane trafficking under co-expression conditions. This finding is compatible with intermolecular interference between NIS molecules, potentially related to NIS oligomerization, but it does not prove a direct dominant-negative mechanism; alternative explanations such as competition for folding or trafficking machinery, altered intracellular retention, indirect effects on plasma membrane delivery, or overexpression-related effects cannot be excluded. Importantly, direct analysis of the patient-derived G288S variant showed a different behavior. In COS-7 cells, WT+G288S co-expression produced an apparent *K_m_* intermediate between those of WT and G288S, with no reduction in *V_max_* compared with WT. Although the *K_m_* increase compared with WT did not reach statistical significance after multiple-comparison correction, the *K_m_* of the co-expression condition differed from that of G288S-expressing cells and showed a numerical rightward shift not observed in MDCK mixed-cell populations, where WT- and G288S-expressing cells were present as separate populations and the apparent *K_m_* remained close to that of WT. Thus, the available data do not support a strong dominant-negative effect of G288S, but they are compatible with a modest co-expression-dependent kinetic impairment. Such a partial reduction in apparent iodide affinity may remain clinically silent under basal conditions but becomes relevant during pregnancy, when iodine requirements and thyroid hormone demand are increased.

Moreover, while NIS dimerization has previously been demonstrated in vitro and proposed to contribute to plasma membrane targeting [14], our findings provide indirect functional support for the pathophysiological relevance of NIS oligomerization in a disease-associated context. Specifically, the selective reduction in WT surface expression observed upon co-expression with a trafficking-defective mutant suggests that interactions between NIS molecules may have direct consequences for plasma membrane delivery and, therefore, for the clinical phenotype. This interpretation is also compatible with previous evidence that NIS can form dimers or higher-order oligomeric states [12].

Taken together, our findings indicate that the patient-derived G288S variant causes an intrinsic kinetic defect characterized by reduced apparent iodide affinity (increased *K_m_*) while preserving plasma membrane targeting and maximal transport capacity. Under heterozygous-like co-expression conditions, G288S does not exert a strong dominant-negative effect, but it may modestly shift iodide transport kinetics toward a lower apparent affinity. In contrast, the severely disruptive G288E substitution provides proof-of-principle evidence that alteration of residue 288 can impair NIS cell-surface delivery under co-expression conditions. Therefore, residue 288 appears to influence NIS function through distinct mechanisms depending on the physicochemical severity of the substitution: reduced apparent iodide affinity in the case of G288S and defective plasma membrane delivery in the case of G288E. More broadly, these findings support the functional relevance of NIS oligomerization for plasma membrane targeting in a disease-associated context.

These observations broaden the clinical spectrum associated with *SLC5A5*/NIS defects and suggest that variants that do not abolish transporter activity under basal conditions may nevertheless become clinically relevant in specific physiological settings, such as pregnancy, resulting in partial ITD phenotypes. Therefore, we do not propose routine *SLC5A5* screening in all women with gestational thyroid dysfunction. However, targeted analysis of *SLC5A5* and other genes related with thyroid dyshormonogenesis may be considered in selected patients with recurrent gestational hyperthyrotropinemia or hypothyroidism, negative thyroid autoimmunity, recovery of thyroid function after pregnancy, or familial clustering of thyroid abnormalities, goiter, or other features suggesting impaired adaptation to increased iodine requirements. Identification of heterozygous *SLC5A5* variants may support closer preconception and early-pregnancy monitoring of TSH and FT4, assessment and optimization of iodine status, and early levothyroxine treatment or dose adjustment when clinically indicated. In addition, although neonatal screening was normal in the offspring of the index patient, previous reports of transient congenital hypothyroidism or transient congenital hyperthyrotropinemia in individuals carrying *SLC5A5* variants suggest that neonatal and early-life thyroid function may represent another relevant window for detecting partial NIS defects when abnormal thyroid function, transient hyperthyrotropinemia, or goiter is present [42]. Rapid growth or puberty could theoretically have a similar effect by increasing thyroid hormone requirements, although this remains speculative and was not documented in the present family.

## 4. Materials and Methods

### 4.1. Ethical Approval and Informed Consent

All individuals involved in the genetic studies provided written informed consent for participation. Written informed consent also covered the use of anonymized clinical and genetic data for scientific publication. The study protocol was reviewed and approved by the Local Research Ethics Committee (CEIm) of La Paz University Hospital, Madrid, Spain, under approval code HULP: PI/1496 (approved on 29 May 2013).

### 4.2. Sequencing

Genomic DNA was isolated from peripheral blood leukocytes using the QIAamp DNA Blood Mini Kit (Qiagen, Hilden, Germany), according to the manufacturer’s instructions. The coding regions and exon–intron boundaries of *SLC5A5* were analyzed using an in-house designed Next-Generation Sequencing (NGS) panel that includes 320 thyroid-related genes and sequenced on a NextSeq 500 Sequencing System (Illumina, Inc., San Diego, CA, USA) [23]. Candidate variants identified by NGS were confirmed by Sanger sequencing.

Sequence alignment, variant calling, and annotation were performed using the GRCh38/hg38 genome reference assembly.

### 4.3. In Silico Prediction

The potential functional impact of the p.Gly288Ser variant was assessed using SIFT (https://www.sift-dna.org/ (accessed on 2 June 2026)), PolyPhen-2 (http://genetics.bwh.harvard.edu/pph2/ (accessed on 2 June 2026)), and CADD v1.6 (https://cadd.bihealth.org/ (accessed on 2 June 2026)). Predictions were obtained through the Ensembl Variant Effect Predictor (VEP) and the CADD web interface using the GRCh38 reference assembly.

Predefined thresholds for pathogenicity were as follows: SIFT score < 0.05 was considered deleterious; PolyPhen-2 score > 0.909 was considered probably damaging, 0.446–0.909 was considered possibly damaging, and ≤0.446 was considered benign; and CADD PHRED score > 20 was considered supportive of deleteriousness effects.

### 4.4. Structural Analysis of NIS

Structural analysis was based on the rat NIS cryo-electron microscopy (cryo-EM) structure with 3.1 Å resolution reported by Ravera et al. [13]. Structural information was retrieved from the Protein Data Bank (PDB ID: 7UV0). This structure, derived from Rattus norvegicus, represents the highest-resolution NIS structure currently available. The sequence identity between the human NIS and the rat template was 86.32%. Structural visualization, hydrophobicity surface rendering, and residue mapping were performed using the PyMOL server (version 3.1.5.1, Schrödinger, LLC, New York, NY, USA). This analysis was used to assess the structural location of residue 288 relative to the transport core and ion-binding regions in human NIS.

### 4.5. Cell Culture and Transfections

COS-7 cells (CVCL_0224), a fibroblast-like African green monkey kidney cell line, and MDCK II cells (CVCL_0424), a polarized epithelial canine kidney cell line, were originally obtained from the American Type Culture Collection (ATCC, Manassas, VA, USA) and maintained in our laboratory. Cells were cultured in Dulbecco’s Modified Eagle Medium (DMEM, Corning, NY, USA) supplemented with 10% FBS (Sigma-Aldrich, St. Louis, MO, USA), 2 mM L-glutamine, and 100 U/mL penicillin and streptomycin (Lonza, Verviers, Belgium) in a humidified incubator at 37 °C with 5% CO_2_. The cell lines were routinely monitored for contamination, including regular testing for mycoplasma. COS-7 cells were transfected with 5 µg of WT or mutant NIS cDNA using Lipofectamine^TM^ 3000 Reagent (Invitrogen, Carlsbad, CA, USA), following the manufacturer’s instructions. Transfection efficiency was assessed by flow cytometry. All experiments were performed 48 h after transfection. For co-expression experiments, equal amounts of WT and mutant plasmids were transfected while keeping the total amount of DNA constant across conditions using an empty plasmid.

MDCK cells stably expressing WT or G288S human NIS were generated by transfection cells with the corresponding constructs inserted into the pcDNA3.1(-)-HA plasmid followed by antibiotic selection with 1 mg/mL Geneticin™ (Thermo Fisher Scientific, Waltham, MA, USA). Individual resistant colonies were isolated using cloning rings, expanded, and screened for NIS expression by immunoblotting prior to functional characterization.

### 4.6. Site-Directed Mutagenesis

Amino acid substitutions at position 288, p.Gly288X, were introduced using the Phusion Site-Directed Mutagenesis Kit (Thermo Fisher Scientific, Waltham, MA, USA). The following oligonucleotides were used: forward 5′-AACCAGGTCVVCCTGTTCCTGA-3′ (where each V can be changed by G+A+C) and reverse 5′-GATGAGCAGGGCCAGCTTG-3′. Substitutions were introduced into the plasmid pcDNA 3.3(-)-HA-hNIS, which carries an HA tag sequence at the N-terminal region of the human NIS protein. Constructs were verified by sequencing, and mutant plasmids were obtained using the GenElute HP Plasmid Midiprep Kit (Sigma-Aldrich, St. Louis, MO, USA) after transformation into *E. coli* DH5α competent cells.

### 4.7. Immunoblot Analysis

Protein lysates (20–40 µg) were prepared in RIPA buffer (1× PBS, 0.1% *v*/*v* SDS, 0.5% *v*/*v* sodium deoxycholate, 1% *v*/*v* Nonidet P-40, and 1% *v*/*v* protease inhibitor cocktail, pH 7.4). The samples were diluted 1:2 in loading buffer, denatured at 90 °C for 5 min, and subjected to 9% SDS-PAGE, followed by transfer to pre-activated PVDF membranes. Immunoblot analysis was carried out with rabbit anti-hNIS-ETNL antibody (1:1000) and rabbit anti-HA antibody (1:1000; Invitrogen, Carlsbad, CA, USA), and HRP-conjugated anti-rabbit IgG secondary antibody (1:2000; Invitrogen, Carlsbad, CA, USA). Incubations were performed overnight at 4 °C, and protein bands were visualized using enhanced chemiluminescence and an Amersham Imager 680 system (Cytiva, Marlborough, MA, USA).

### 4.8. Immunofluorescence

Transfected cells were grown on glass coverslips, washed twice with PBS, and incubated with Wheat Germ Agglutinin (WGA) conjugated to Alexa Fluor™ 647 (1:200; Invitrogen, Carlsbad, CA, USA) for 15 min. The cells were then washed again and fixed with 4% paraformaldehyde for 15 min at room temperature. Two conditions were established: (1) permeabilized, in which cells were treated for 5 min with a permeabilization buffer consisting of 0.5% Triton X-100 and 2% BSA in PBS; and (2) non-permeabilized, in which all steps were performed with 2% BSA in PBS. Blocking was performed for 1 h at room temperature in a humidified chamber (0.05% Triton X-100 and 2% BSA in 1× PBS; without Triton for non-permeabilized samples).

Coverslips were incubated with a mouse anti-HA primary antibody (1:200; Invitrogen, Carlsbad, CA, USA) for 1 h, washed twice with PBS, and incubated with Alexa Fluor^®^ 488-conjugated goat anti-mouse IgG secondary antibody (1:5000; Invitrogen, Carlsbad, CA, USA). Both primary and secondary antibodies were diluted in PBS (non-permeabilized) or PBS-Triton X-100 (permeabilized). After three washes, nuclei were stained with DAPI (1:5000) for 15 min. The coverslips were mounted using ProLong™ Glass Antifade Mountant (Invitrogen, Carlsbad, CA, USA) and stored at 4 °C in the dark. Imaging was performed using a Leica SP5 confocal microscope, and images were processed with LAS X software v1.4.7.28982 (Leica Microsystems, Wetzlar, Germany).

### 4.9. Flow Cytometry

For each condition (permeabilized and non-permeabilized), 5 × 10^5^ transfected cells were selected. For permeabilized samples, cells were incubated with Fixation/Permeabilization solution (BD Biosciences, San Jose, CA, USA) for 20 min, and then washed with Perm/Wash solution with centrifugation at 1000 rpm for 5 min at 4 °C (all washes were performed under these centrifugation conditions). For non-permeabilized cells, PBSA (PBS pH 7.4 with 0.1% BSA) was used instead.

The cells were incubated with rabbit anti-HA primary antibody (1:200; Invitrogen, Carlsbad, CA, USA) for 1 h at 4 °C, followed by Alexa Fluor^®^ 488-conjugated goat anti-rabbit secondary antibody (1:2000; Invitrogen, Carlsbad, CA, USA) for 1 h at room temperature in the dark. The antibodies were diluted in PBSA (non-permeabilized cells) or Perm/Wash (permeabilized cells). After washing, the cells were resuspended in PBS and analyzed using a BD FACSCanto flow cytometer. Data were analyzed with BD FACS Diva and FlowJo v10.10 software.

### 4.10. ^125^I^−^ Transport Assays

Iodide uptake was measured in transfected cells. Cells were washed twice with Hank’s Balanced Salt Solution (HBSS, Corning, USA) and incubated in HBSS containing 20 µM KI, 140 mM NaCl, and 2 mM MMI, supplemented with 1 µCi carrier-free Na^125^I^−^ to give a specific activity of 100 µCi/mmol. For steady-state experiments, cells were incubated in the presence of 20 µM I^−^/140 mM Na^+^ or 20 µM I^−^/140 mM Na^+^/80 µM ClO4^−^ for 1 h at 37 °C, and then washed twice with ice-cold HBSS and lysed with 500 µL of cold ethanol for 20 min at 4 °C. Radioactivity was quantified using a Ƴ-counter (Packard Cobra, Packard Instrument Inc., Downers Grove, IL, USA). DNA content was measured by the diphenylamine method after trichloroacetic acid precipitation. Uptake was expressed as picomoles of I^−^ per µg of DNA. The results represent the mean ± SEM of at least three independent experiments performed in triplicate. The data were normalized to WT NIS activity within each experiment. Statistical significance was determined using unpaired two-tailed Student’s *t*-test, and differences were considered significant if *p* ≤ 0.05.

For I^−^-dependent kinetics analysis, cells were incubated with the indicated concentrations of iodide (1–300 µM) and 140 mM NaCl for 4 min. This iodide concentration range was selected to allow for robust estimation of apparent kinetic parameters, including *K_m_* and *V_max_*, rather than to reproduce a single physiological plasma iodide concentration. Lower micromolar concentrations were included to assess uptake under low-iodide conditions, whereas higher concentrations were required to approach transporter saturation. Initial rate data were analyzed by nonlinear regression using the Michaelis–Menten equation: *v*([I^−^]) = (*V_max_* × [I^−^])/(*K_m_* + [I^−^]). Uptake values from non-transfected cells were subtracted as background prior to analysis. Kinetic parameters are reported as the mean ± SD from at least three independent experiments. For Na^+^-dependent kinetics analysis, cells were incubated with varying Na^+^ concentrations (0–240 mM) in the presence of 20 µM I^−^ for 4 min; the osmolarity was adjusted using choline. The data were analyzed using an allosteric sigmoidal model, with the Hill coefficient (*n_H_*) as a variable parameter and V_0_ fixed at 0. Three independent experiments were included in this joint analysis (one using COS-7 cells and two using MDCK cells).

To evaluate the potential functional influence of the G288S variant on WT NIS, iodide transport assays were performed using mixed populations of MDCK cells expressing either WT or G288S NIS. A 1:4 WT:G288S ratio was used to compensate for the lower NIS expression levels observed in the mutant clone compared with the WT clone. Steady-state assays and I^−^-dependent kinetics analysis were then performed as described above. Transport activity was normalized according to the NIS expression levels determined by Western blot analysis.

### 4.11. Sequence Analysis

Representative NIS sequences from different species were obtained from UniProt and aligned using Clustal Omega v1.2.4 (European Bioinformatics Institute, Hinxton, Cambridge, UK). The UniProt ID numbers of the sequences used were Q92911 (*Homo sapiens*), A4IG60 (*Danio rerio*), Q99PN0 (*Mus musculus*), Q63008 (*Rattus norvegicus*), F1MT46 (*Bos taurus*), A0A8D0YYR1 (*Sus scrofa*), H2LQI7 (*Oryzias latipes*), A0A4W3GYU6 (*Callorhinchus milii*), Q6DDF5 (*Xenopus laevis*), H3B2N3 (*Latimeria chalumnae*), A0A8V1AH69 (*Gallus gallus*), A0AAJ7X981 (*Petromyzon marinus*), and A0A8C0P3J1 (*Canis lupus familiaris*).

## Data Availability

The original contributions presented in this study are included in the article. Further inquiries can be directed to the corresponding author.

## Abbreviations

The following abbreviations are used in this manuscript:

AP-1	Adaptor protein 1
BSA	Bovine serum albumin
CH	Congenital hypothyroidism
ClO4^−^	Perchlorate
cryo-EM	Cryo-electron microscopy
DMEM	Dulbecco’s Modified Eagle Medium
DMSO	Dimethyl sulfoxide
DAPI	4′,6-diamidino-2-phenylindole
FACS	Fluorescence-activated cell sorting
FBS	Fetal bovine serum
FT4	Free thyroxine
HA	Hemagglutinin
HBSS	Hank’s Balanced Salt Solution
hNIS	Human sodium/iodide symporter
HRP	Horseradish peroxidase
I−	Iodide
IDD	Iodine deficiency disorder
ITD	Iodide transport defect
KI	Potassium iodide
LT4	Levothyroxine
MDCK-II	Madin–Darby canine kidney II
MMI	Methimazole
Na+	Sodium
NIS	Sodium/iodide symporter
PBS	Phosphate-buffered saline
PBSA	Phosphate-buffered saline containing bovine serum albumin
PCR	Polymerase chain reaction
PDB	Protein Data Bank
PM	Plasma membrane
SD	Standard deviation
SEM	Standard error of the mean
*SLC5A5*	Solute carrier family 5 member 5
TH	Thyroid hormone
TMS	Transmembrane segment
TSH	Thyroid-stimulating hormone
VEP	Variant Effect Predictor
VUS	Variant of uncertain significance
WGA	Wheat germ agglutinin
WT	Wild type
